# The efficacy and safety profile of albumin administration for patients with cirrhosis at high risk of hepatorenal syndrome is dose dependent

**DOI:** 10.1093/gastro/gov032

**Published:** 2015-07-15

**Authors:** Yuliya Afinogenova, Elliot B. Tapper

**Affiliations:** ^1^Harvard Medical School, Boston, MA, USA, and; ^2^Division of Gastroenterology and Hepatology, Beth Israel Deaconess Medical Center, Harvard Medical School, Boston, MA, USA

**Keywords:** albumin, cirrhosis, ascites, hepatorenal syndrome, acute renal failure, spontaneous bacterial peritonitis

## Abstract

**Background:** Albumin is a critical component in the standard therapeutic approach to acute renal failure (ARF) and spontaneous bacterial peritonitis (SBP) in the setting of ascites. However, data regarding the safety and minimum effective dose are limited.

**Methods:** We conducted a retrospective review of patients with decompensated cirrhosis who received albumin within the first 48 hours of hospitalization at Beth Israel Deaconess Medical Center between 2010 and 2013. Outcomes included 90-day risk of death or transplantation (primary) and (secondary) complications of albumin infusion (length of stay (LOS) and need for critical care)), all adjusted for comorbidity and severity of illness.

**Results:** We included 169 patients with ARF and 88 patients with SBP. The optimal doses of albumin for a survival benefit were found to be 87.5 g and 100 g in the ARF and SBP cohorts, respectively. The odds ratio (OR) for the 90-day risk of death or liver transplantation associated with the optimal loading dose was 0.36 (95% CI: 0.17–0.76, *P* = 0.008) and 0.28 (95% CI: 0.07–0.97, *P* = 0.04) for the ARF and SBP cohorts, respectively. This effect persisted for patients with ARF who had neither hepatorenal syndrome (HRS) nor SBP (OR: 0.13, 95% CI: 0.007–0.79, *P = *0.02). LOS (beta coefficient per log albumin dose: 1.69; 95% CI: 0.14–3.24, *P* = 0.03) and risk of critical care (OR/g albumin: 1.03; 95% CI: 1.01–1.05, *P* = 0.01) were also dose dependent.

**Conclusion:** Albumin has a dose-dependent effect on both survival and complications in patients with cirrhosis with ARF (HRS and otherwise) and/or SBP.

## Introduction

Acute renal failure (ARF) and spontaneous bacterial peritonitis (SBP) are important sources of morbidity and mortality in patients with cirrhosis. Patients with cirrhosis complicated by ascites who develop any form of renal failure have survival rates of approximately 50% at one month and 20% at six months [1]. SBP is associated with non–infection-related in-hospital mortality rates of 20%–40% [2, 3] and respective one- and two-year mortality rates up to 70% and 80% without transplantation [4–8]. Indeed, renal failure is the major determinant of survival in SBP. In a modern cohort, 30%–40% of patients with SBP who develop concomitant renal failure are those at the highest risk of death. Renal failure complicating SBP is associated with a mortality rate of 67%, compared with 11% among those without concomitant renal failure [9].

Standard treatment of ARF and SBP includes albumin infusion [10]. Cirrhotic hemodynamics can easily lead to arterial underfilling. For this reason, an ‘albumin challenge’ at doses of 1 g/kg /day for at least 2 days are recommended to exclude hypovolemic renal failure and to diagnose hepatorenal syndrome (HRS) [11]. Thereafter, albumin is also recommended for the treatment of type 1 HRS, albeit with doses that vary significantly across studies [12–16]. Similarly, current guidelines recommend that all patients with SBP be treated with albumin at doses of 1.5 g/kg on day one and 1 g/kg on day three to forestall the development of HRS [17].

However, for clinicians interested in improving the quality of care provided to patients with ascites, many questions remain regarding the use of albumin. First, the benefits of albumin in low-risk SBP patients continue to be debated [18–21]. Second, the minimum effective albumin dose needed to prevent renal failure in SBP has not been established. Indeed, optimal dosing is important, given the cost of albumin [22] as well as the potential for complications of volume overload such as respiratory distress [23, 24]. Finally, the optimal dose of albumin in patients with acute creatinine elevations for prevention of HRS has not been explored [12–16].

Herein, we explore the determinants of the efficacy and safety of albumin infusion in a cohort of patients with ascites who present at high risk for HRS in an American liver transplant center.

## Methods

We performed a retrospective cohort study of patients with cirrhosis at Beth Israel Deaconess Medical Center in Boston. The study took place on a liver transplant unit with an average of 600 annual admissions. Criteria for admission to this service included an established diagnosis of decompensated cirrhosis or a medically complicated liver transplant. All clinical care was provided on the dedicated inpatient hepatology unit, which was staffed by house staff and a hepatologist. No changes in the number of staff, nursing and house staff occurred during the time of the study. This study was conducted in accordance with the Declaration of Helsinki and was approved by our institutional review board. The cohort design and analysis of this study were performed consistent with STROBE (Strengthening the Reporting of Observational Studies in Epidemiology) guidelines [25].

### Collection of Data

**Figure 1.**
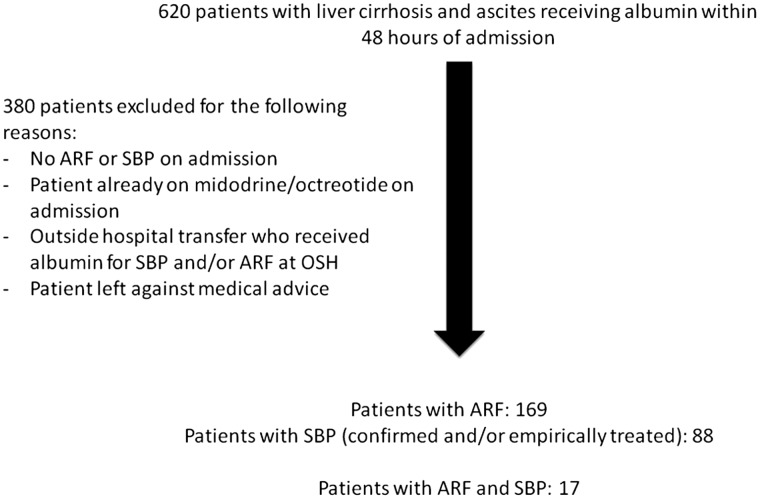
Exclusion criteria. ARF = acute renal failure, SBP = spontaneous bacterial peritonitis, OSH = outside hospital.

All patients (N = 620) with cirrhosis and ascites who were admitted to the hospital between 2010 and 2013 and who received albumin within the first 48 hours of admission for any indication were screened for the study. Patients who did not have a diagnosis of ARF or SBP on admission (as interpreted from their discharge summaries) were excluded. The complete exclusion criteria are shown in Figure 1. Thereafter, patients were categorized within one or both of two cohorts defined by the presence of ARF or SBP. ARF was defined by an admission creatinine ≥ 1.3 mg/dL, which was elevated from baseline by > 0.3 mg/dL. Patients had different etiologies of ARF. Since HRS is a clinical diagnosis and this study took place outside the trial setting, patients were defined as having HRS by either meeting the International Club of Ascites criteria [26] or by implementation of early therapy (identified by initiation of midodrine) in which high clinical suspicion affected clinical management. The presence of SBP was defined by a paracentesis on admission, with an ascitic fluid cell count containing > 250 neutrophils or documented high clinical suspicion for SBP requiring empiric treatment. Patients started on therapy without a diagnostic paracentesis were included to more effectively model a real-world practice in which antibiotics are often administered prior to paracentesis. Patients with SBP received either empiric antibiotic therapy or directed therapy when the organism could be identified.

The primary outcome was the risk of death within 90 days or transplant on current admission. Mortality data are complete as confirmed using a validated online search of the United States Social Security Death Index [27]. Secondary outcomes included hospital length of stay (LOS), floor-to-intensive care unit (ICU) transfer following albumin infusion and transfer to ICU for fluid overload.

The principle exposure variables studied were grams of albumin administered at the first dose and the time from admission to the start of albumin infusion. Of note, if the patient received a second dose of albumin within 12 hours of the first dose, the combined dose was reported, and the time was calculated to the initiation of the first dose.

Covariates for regression analyses included age, sex, Charlson comorbidity index, Model for End-stage Liver Disease (MELD score, sodium level on admission and administration of beta blockers at the time of albumin order [28]. The Charlson comorbidity index was calculated using ICD-9 codes, as previously described [29]. The MELD score was calculated using the United Network for Organ Sharing (UNOS) modification according to previously described algorithms [30].

### Data Analysis

JMP Pro statistical discovery software (version 11) was used for statistical analyses. Subject characteristics and outcome variables were reported in the ARF and SBP cohorts independently. Data were summarized as mean ± standard deviation for normally distributed, median and interquartile range (IQR) for non-normally distributed continuous outcomes or counts and percentages for categorical outcomes. A two-tailed *P*-value was considered significant when < 0.05.

Logistic regression was performed to assess associations with the binary outcomes (death or transplantation, ICU transfer). All multivariate analyses were adjusted for age, sex, Charlson comorbidity index and the covariates that were significantly associated with outcome variables in the univariate analyses. A *P*-value < 0.10 was required for inclusion in the multivariate model. We also used the receiver operating characteristic curves to determine the optimal loading dose of albumin for a mortality benefit in both the ARF and SBP cohorts. For 90-day risk of death or transplantation during hospitalization, odds ratios (ORs) were reported per gram of albumin administered and for whether or not at least an optimal dose was administered. For ICU transfer, the OR was reported per gram of albumin administered. For the univariate and multivariate regressions for the LOS variable, we treated the outcome as a continuous variable and used a linear regression. As the LOS has a right skew, it cannot be dichotomized or legitimately assessed using a logistic regression. Thus, we used a negative binomial generalized regression. The output of the analysis is a beta coefficient for the LOS, which indicates an increase in the LOS for every log increase in the independent variable. The multivariate regressions for the LOSs were performed according to the specifications above. For LOS, beta coefficient is reported per gram of albumin administered.

For the ARF cohort, we also repeated the analyses excluding HRS and SBP patients as a sensitivity analysis to assess the benefit of albumin in a lower-risk subset. We determined OR for 90-day risk of death or transplantation on hospitalization per gram of albumin administered and for whether or not at least an optimal dose was administered. Subsequently, we also examined the reasons for floor-to-ICU transfers in the entire ARF cohort through chart reviews and determined whether the reason for the ICU admission was related to fluid overload secondary to albumin infusion.

## Results

Clinical characteristics and demographics of patients in the ARF and SBP groups are described in Table 1. By design, all patients were Child-Pugh class B or C with variable MELD, as delineated in Table 1. Patients in the ARF cohort were sicker overall, with higher Charlson comorbidity indexes, MELD scores and creatinine levels. An average loading dose of 77.34 ± 28.48 g of albumin was administered within a median of 4 hours (IQR: 1–13) to patients in the ARF cohort following registration in the emergency department. Patients with SBP received an average loading dose of 108.58 ± 35.33 g of albumin within a median of 4 hours (IQR: 1–9.5) post admission. Seventy out of 169 (41.42%) patients in the ARF cohort and 23 out of 88 (26.14%) patients in the SBP cohort were clinically diagnosed with HRS. The risk of death at 90 days or transplantation during hospitalization was equivalent for patients with ARF and SBP.

**Table 1.** Subject characteristics and demographics

Characteristics	ARF cohort (*n* = 169)	SBP cohort (*n* = 88)
Age, years	58.33 ± 10.90	55.75 ± 12.52
Sex, *n* (% male)	115 (68.05)	54 (61.36)
Race, *n* (% white)	120 (71.01)	62 (70.45)
Etiology of cirrhosis, *n* (%)		
Alcohol	46 (27.22)	18 (20.46)
Hepatitis C	53 (31.36)	32 (36.36)
Alcohol and hepatitis C	22 (13.01)	14 (15.91)
Other	48 (28.40)	24 (27.27)
Past or present HCC diagnosis, *n* (%)	19 (11.24)	11 (12.50)
Etiology of renal failure, *n* (%)		
Prerenal azotemia	78 (46.15)	–
Hepatorenal syndrome type 1	72 (42.60)	–
Acute tubular necrosis	6 (3.55)	–
Acute interstitial nephritis	4 (2.37)	–
Contrast induced	0 (0)	–
Other*	9 (5.33)	–
Charlson Comorbidity Index	5.67 ± 2.53	4.91 ± 2.23
Admission MELD Score	24.95 ± 6.88	21.59 ± 7.50
Sodium, mEq/L	131.44 ± 7.07	131.38 ± 7.00
Creatinine, mg/dL	2.46 ± 1.03	1.44 ± 0.92
Total bilirubin, μmol/L	3.6 (1.4–6.3)	3.9 (1.9–9.1)
Midodrine/octreotide at discharge, *n* (%)	70 (41.42)	23 (26.14)
beta blockaders at time of albumin, *n* (%)	47 (28.14)	29 (32.95)
Time to albumin, hours	4 (1–13)	4 (1–9.5)
First dose of albumin, grams	77.34 ± 28.48	108.58 ± 35.33
**Primary and secondary outcomes**		
Transplanted on current admission, *n* (%)	15 (8.88)	6 (6.82)
Expired at 90 days or transplanted during hospitalization, *n* (%)	64 (37.87)	31 (35.23)
Length of stay, days	6 (3.5–13.5)	7 (4.0–11.8)
Floor-to-ICU transfer after albumin, *n* (%)	30 (17.75)	13 (14.77)

Continuous values presented as mean ± standard deviation or median (interquartile ranges).

ARF=acute renal failure. HCC=hepatocellular carcinoma. MELD=Model for End Stage Liver Disease. SBP=spontaneous bacterial peritonitis.

* Three patients with components of prerenal and intrinsic renal disease, three patients with components of prerenal and postrenal disease, one patient with tacrolimus toxicity, one patient with type II HRS, one patient with membranoproliferative glomerulonephritis and prerenal disease.

Table 2 presents the associations of clinical predictors with the 90-day risk of death or transplantation on hospitalization in univariate and multivariate analyses for ARF and SBP cohorts. The outcome was associated with the loading dose of albumin and MELD score in both cohorts. In an analysis of the receiver operating characteristics, the optimal dose of albumin for a mortality benefit was 87.5 g and 100 g in the ARF and SBP cohorts, respectively. Adjusted for the MELD, the ORs for mortality associated with these optimal doses were 0.36 (95% CI: 0.17–0.76, *P* = 0.008) and 0.28 (95% CI: 0.07–0.97, *P* = 0.04) for the ARF and SBP cohorts, respectively. Adjusted for the MELD, the ORs per gram albumin for mortality were 0.98 (95% CI: 0.97–0.99, *P* = 0.007) and 0.98 (95% CI: 0.96–0.99, *P* = 0.049) for the ARF and SBP cohorts, respectively. In a sensitivity analysis on the subset of 90 patients with ARF who had neither HRS nor SBP. The adjusted dose effect of albumin persisted for this group, OR for the optimal albumin dose 0.13 (95% CI: 0.007–0.79, *P* = 0.02) and OR per gram albumin 0.98 (95% CI: 0.96–0.99, *P* = 0.03).

**Table 2.** Univariate and multivariate analyses for determinants of 90-day risk of death or transplant during hospitalization in acute renal failure and spontaneous bacterial peritonitis cohorts

Variables	Acute Renal Failure cohort						Spontaneous Bacterial Peritonitis cohort					
	Univariate analysis			Multivariate analysis			Univariate analysis			Multivariate analysis		
	OR	95% CI	*P*-value	OR	95% CI	*P*-value	OR	95% CI	*P*-value	OR	95% CI	*P*-value
Albumin, grams	0.99	0.98–1.00	0.079	0.98	0.97–0.99	0.007	0.98	0.72–0.99	0.01	0.98	0.96–0.99	0.049
Age, years	1.01	0.98–1.03	0.67				1.04	1.00–1.09	0.03	1.08	1.03–1.15	0.001
Sex, male	1.50	0.77–2.90	0.23				1.87	0.77–4.63	0.17			
Charlson Comorbidity Index, per unit	1.01	0.89–1.15	0.81				1.15	0.94–1.43	0.17			
MELD score, per unit	1.12	1.07–1.18	<0.001	1.17	1.10–1.26	<0.001	1.13	1.06–1.22	<0.001	1.19	1.08–1.32	0.001
Sodium, mEq/L	0.97	0.93–1.02	0.21				0.91	0.83–0.98	0.03	0.90	0.79–0.99	0.05
Beta blockade*	1.47	0.73–3.06	0.28				2.16	0.82–6.17	0.13			
Time to albumin, hours	1	0.96–1.05	0.99				1.02	0.97–1.09	0.08			

Variables with *P* < 0.10 were included in the multivariate analysis as covariates. Regardless of significance, the following variables were also included in the multivariate analysis: age, sex and Charlson comorbidity index.

OR=odds ratio. CI=confidence interval. MELD=Model for End Stage Liver Disease.

* at the time of albumin administration.

Table 3 presents the associations of clinical predictors with the length of hospital stay and the probability of floor-to-ICU transfer in the ARF group in univariate and multivariate analyses. LOS was associated with the dose of albumin, MELD, sodium, beta blockade at time of albumin administration and time to albumin in the ARF cohort. Adjusting for factors significant in the univariate analysis, the beta coefficient per grams of albumin administered was 1.69 (95% CI: 0.14–3.24, *P* = 0.03). This indicates that for every log increase in the albumin dose, LOS increased by 1.69 days. The probability of floor-to-ICU transfer was associated with the dose of albumin, sex, MELD, sodium and whether or not the patient was transplanted during the admission. Adjusting for factors significant in the univariate analysis, the OR per gram albumin for floor-to-ICU transfer was 1.03 (95% CI: 1.01–1.05, *P* = 0.01). In the SBP cohort, the dose of albumin was not associated with the length of hospital stay and the probability of floor-to-ICU transfer.

**Table 3.** Univariate and multivariate analyses for determinants of length of hospital stay and floor-to-ICU transfer risk following albumin administration in the acute renal failure cohort

Variables	Length of hospital stay						Risk of floor-to-ICU transfer during admission					
	Univariate analysis			Multivariate analysis			Univariate analysis			Multivariate analysis		
	Beta**^#^**	95% CI	*P*-value	Beta**^#^**	95% CI	*P*-value	OR	95% CI	*P*-value	OR	95% CI	*P*-value
Albumin, grams	3.48	1.70–5.26	<0.001	1.69	0.14–3.24	0.03	1.02	1.01–1.04	0.003	1.03	1.01–1.05	0.01
Age, years	−1.90	−3.62– −0.18	0.03	0.54 95%	0.10–3.15	0.50	1.03	0.99–1.07	0.11			
Sex, male	3.97	2.29–5.65	<0.001	2.99 95%	1.48–4.49	<0.0001	4.29	1.90–10.00	<0.001	2.73 95%	0.56–13.2	0.21
Charlson Comorbidity Index, per unit	0.58	−1.55–2.71	0.59				1.01	0.85–1.17	0.94			
MELD Score, per unit	6.29	4.65–7.92	<0.001	4.78	3.08–6.50	<0.001	1.18	1.11–1.27	<0.001	1.20	1.10–1.33	<0.001
Sodium, mEq/L	−4.40	−6.26– −2.52	<0.001	−2.16	−3.79– −0.52	0.01	0.93	0.87–0.98	0.01	0.97	0.99–1.05	0.47
Beta blockade*	2.93	1.16–4.72	0.002	0.29	−1.28–1.86	0.72	1.35	0.56–3.64	0.52			
Time to albumin, hours	−1.80	−3.58– −0.02	0.048	-0.41	−1.88–1.06	0.60	1.00	0.95–1.07	0.86			
Transplantation during admission	–	–	–				4.98	1.61–15.24	0.006	4.60	0.98–22.51	0.05

Variables with *P* < 0.10 were included in the multivariate analysis as covariates. Regardless of significance, the following variables were also included in the multivariate analysis: age, sex and Charlson comorbidity index.

ICU = Intensive care unit. OR = odds ratio. CI = confidence interval. MELD = Model for End Stage Liver Disease.

* at the time of albumin administration.

**^#^** Note that the beta coefficient represents the increase in length of stay (days) for every log increase in exposure variable.

Table 4 examines the reasons for floor-to-ICU transfers that occurred for ARF and SBP cohorts independently. Eight out of 30 (27%) patients with ARF were transferred to the ICU for reasons related to volume overload, such as pulmonary edema or initiation of continuous venovenous hemofiltration (CVVH). Five additional patients were transferred with gastrointestinal bleeding (with the sources of bleeding identified in the Table 4 legend), some of which may be related to fluid overload. Only one out of 11 (9%) patients with SBP was transferred to the ICU due to causes related to fluid overload (pulmonary edema in this case); however, this patient also had impaired renal function.

**Table 4.** Reasons for floor-to-ICU transfers after albumin administration in patients with acute renal failure and spontaneous bacterial peritonitis

Reasons	ARF cohort (n = 169)	SBP cohort (n = 88)
Respiratory distress, n (%)	10 (5.9)	2 (2.3)
Gastrointestinal bleed, n (%)	5 (3.0)*	0 (0)
CVVH, n (%)	1 (0.6)	0 (0)
Encephalopathy, n (%)	4 (2.4)	0 (0)
Hypotension, n (%)	4 (2.4)	3 (3.4)
Post transplant, n (%)	6 (3.6)	2 (2.3)
Other, n (%)	0 (0)	4 (4.5)

CVVH = continuous veno-venous hemofiltration.

*Two patients with confirmed or suspected variceal bleeding, two with non-variceal upper gastrointestinal bleeding and one with lower gastrointestinal bleeding.

## Discussion

To date, the use of albumin has been widely accepted for the following three indications: management of HRS, prevention of renal failure after SBP and prevention of renal injury following large volume paracentesis; however, many questions regarding the efficacy and safety of albumin remain. This study of albumin infusion for patients with decompensated cirrhosis and ARF or SBP demonstrated that albumin has a dose-dependent effect on survival in both patients with ARF and SBP. Additionally, albumin infusion is associated with infrequent but significant harms related to fluid overload that are also dose-dependent, particularly in patients with renal impairment.

Our data confirm and extend our current knowledge on the role of albumin in four ways. First, in this large cohort study, we confirmed that albumin infusion reduces 90-day mortality or the risk of transplant for patients with ARF and established that this survival benefit holds even for patients with ARF attributable to causes other than HRS.

Second, while it is well established that albumin, along with vasoconstrictors, is efficacious for reducing mortality in patients with type 1 HRS [14,15,31], the optimal loading dose is unclear. We now show that the survival benefit appears to be dose-dependent, with an optimal dose of 87.5 g in the loading phase. The dose-dependent benefits of albumin hold for patients with HRS and non-HRS renal failure. Similarly, while 1.5 g/kg of albumin is recommended for patients with SBP [18], we confirmed the mortality benefit of patients with SBP and determined an optimal dose (100 grams).

Third, despite its positive effects on survival, there was a simultaneous dose-dependent association between albumin and both prolonged hospital stay and increased risk of ICU transfer, particularly for fluid overload, in patients with ARF. These data highlight the need for careful observation and selection of albumin doses that consider the renal and cardiac status of each individual patient.

Pulmonary edema and volume overload following albumin infusion have been reported previously. Two prior studies investigating the efficacy of albumin for non-SBP infections have previously suggested the risk of pulmonary edema. Guevara *et al*.** and Thevenot *et al*.** identified three (5%) and eight (8%) patients who developed pulmonary edema in their respective studies of albumin for non-SBP infections [23,24]. In their recent landmark trial of terlipressin for hepatorenal syndrome, Cavallin *et al.* described two (4%) patients with ‘circulatory overload’ following a protocol of albumin at 1 g/kg on day one followed by 20–40 g/day thereafter [16]. Our study confirms the risk of fluid overload in a large cohort of unselected patients outside the context of a clinical trial. Furthermore, we show that the rate (4.5%) is similar to prior studies [16] and essentially exclusive to the cohort presenting with acute renal impairment. Furthermore, we show that the actual loading dose of albumin is directly related to the risk of adverse events, indicating that doses in excess of 87.5 g should be used with caution. Further study is needed to clarify the optimal dosing strategy in patients with limited renal function.

Lastly, in our study, we were unable to detect an association between time to albumin administration and survival within the first 48 hours of admission. While we cannot exclude the possibility that we were underpowered for detecting an association, these data highlight a role for caution, not urgency in the context of our findings regarding the adverse effects of albumin. Careful examination and consideration of a patient's risk for fluid overload should guide albumin dosing and timing decisions.

Our data must be interpreted within the context of the study design. First of all, this is a single-center study at a transplant center in the USA and is therefore without access to terlipressin; thus, it is unclear whether these results may be generalizable to other settings. We studied the dose of albumin irrespective of weight for several reasons. First, prior studies are inconsistent regarding the loading dose, varying from fixed 10–100 g doses to weight-based dosing [12,15,16]. Second, ideal body weight is usually at significant odds with total body weight in cirrhotic patients with ascites, edema and sarcopenia. As this was a retrospective analysis, ARF was defined as creatinine of at least 1.3 and increased from baseline; however, we did not account for the rate of change in creatinine level as these data were unavailable for most patients. Furthermore, while there were multiple etiologies for ARF in this study, the limited sample size precludes efficient subgroup analyses. Additionally, the definition of HRS was based in part on clinical management including early initiation of midodrine therapy in order to reflect real-world practice. As such, by design, these data apply to daily clinical practice in our center, and whether this is generalizable elsewhere is unknown. Furthermore, this practice may have overestimated the number of patients who developed true HRS by international consensus definitions [26]. Similarly, we included patients with empiric treatment of SBP without ascitic fluid studies to model practice at our institution in order to render data with clearer quality-improvement implications for our clinicians; this may not be generalizable and may have overestimated the number of patients with true SBP. We also included only patients who were admitted with SBP or renal injury, and therefore these data cannot speak to outcomes following the development of nosocomial complications. Finally, we only studied albumin infusions within the first 48 hours; the associations studied are strictly based on the initial dosing decision and did not include the total dose provided.

In conclusion, albumin has a dose-dependent effect on survival in patients with ARF and SBP, with the persistence of the dose effect even for patients with renal failure for reasons other than HRS. Furthermore, albumin also has a dose-dependent effect on complications as assessed by the hospital LOS and risk of ICU admission. Complications associated with fluid overload are particularly worrisome and demand further study to determine the optimal dose of albumin for at-risk patients.

## Funding

Ms. Afinogenova was supported by a grant from the Scholars in Medicine office, Harvard Medical School. Dr. Tapper was supported by a grant from the Carl J. Shapiro Institute for Education and Research. The content is solely the responsibility of the authors and does not necessarily represent their funding institutions.

*Conflict of interest statement*: none declared.
